# Pattern Recognition of Momentary Mental Workload Based on Multi-Channel Electrophysiological Data and Ensemble Convolutional Neural Networks

**DOI:** 10.3389/fnins.2017.00310

**Published:** 2017-05-30

**Authors:** Jianhua Zhang, Sunan Li, Rubin Wang

**Affiliations:** ^1^School of Information Science and Engineering, East China University of Science and TechnologyShanghai, China; ^2^School of Sciences, East China University of Science and TechnologyShanghai, China

**Keywords:** mental workload, pattern classification, convolutional neural network, ensemble learning, deep learning, electrophysiology

## Abstract

In this paper, we deal with the Mental Workload (MWL) classification problem based on the measured physiological data. First we discussed the optimal depth (i.e., the number of hidden layers) and parameter optimization algorithms for the Convolutional Neural Networks (CNN). The base CNNs designed were tested according to five classification performance indices, namely Accuracy, Precision, F-measure, G-mean, and required training time. Then we developed an Ensemble Convolutional Neural Network (ECNN) to enhance the accuracy and robustness of the individual CNN model. For the ECNN design, three model aggregation approaches (weighted averaging, majority voting and stacking) were examined and a resampling strategy was used to enhance the diversity of individual CNN models. The results of MWL classification performance comparison indicated that the proposed ECNN framework can effectively improve MWL classification performance and is featured by entirely automatic feature extraction and MWL classification, when compared with traditional machine learning methods.

## Introduction

With the rapid development of automation technology, automatic control systems have been extensively applied to almost every engineering field. HMS and HMI are necessitated because the design of technologies often does not account for and leverage how humans behave and think. For example, automation has been studied and applied extensively, non-etheless a major observation is that automation systems do not remove humans from the workplace, but instead changes the nature of their tasking and create new coordination demands on human operators (Parasuraman et al., 2000). Some tasks, e.g., air traffic control, still need to be completed by the collaboration or integration between human and automation system. This broad class of systems that include human factors is referred to as Human-Machine System (HMS; Hollender et al., 2010). In a HMS, excessively high MWL levels mean that the tasks required to handle exceed the operators' capacity. The operators cannot respond to the unexpected events even if they can maintain the system well. On the contrary, too low MWL makes it difficult for operators to concentrate on the current tasks.

To prevent such accidents, some researchers argued that it is important to maintain the optimal Operator Functional State (OFS) in human-machine collaborative tasks (Mahfouf et al., 2007). The accurate and computationally efficient OFS assessment and prediction method can be used to adjust the level of automation (or autonomy) in a HM shared control system. A crucial problem is how to estimate the OFS in real time and accurately. Previous studies showed that the MWL is a critical dimension of the OFS construct. The MWL of the human operator would constantly increase once the workload exceeds certain threshold and wrong decision-making or accidents would occur.

There are three major techniques for the MWL assessment: (1) subjective assessment—The assessment results depend on the participants' self-perception of the task and thus are often affected by the interpretations of questionnaire, answering style and memory capacity (Kivikangas et al., 2011). The subjective ratings were performed following each task-load condition in our experiments, they are difficult to apply in real-time applications; (2) task performance based evaluation—This technique overcomes certain shortcomings of the first method. The operator is regarded to be in a better functional state if he exhibits better task performance. The method still has some drawbacks as some well-trained operators may persevere in execution of the tasks with seemingly good performance even if they are overloaded mentally; and (3) psychophysiological data based assessment—Based on an effective fusion of the Electroencephalogram (EEG), Electromyogram (EMG), Electrooculogram (EOG), and Functional Magnetic Resonance Imaging (FMRI) data, this method can characterize the OFS accurately (Hollender et al., 2010). The physiological responses of the operator provide an objective and instant (or real-time) assessment of operator functional state (OFS) in general and the MWL in particular. Nevertheless, the method suffers from two major shortcomings. One disadvantage is concerned with data acquisition and interpretation, that is, the measured psychophysiological signal is usually weak in amplitude and contaminated by various external noises, disturbances or artifacts. Thus, it is important to choose suitable filters to filter out those unwanted components. For example, the EOG signal recording is often affected by blinking. In addition, the measured data are usually large-scale and thus demand sophisticated big data analysis techniques/algorithms. Another disadvantage is the requirement of special-purpose equipment, which is usually expensive and requires high maintenance cost.

In recent years many researchers have focused on the challenging problem of MWL assessment. For example, Parasuraman (Parasuraman and Wilson, 2008) used physiological signals to evaluate the MWL of the operator in unmanned aerial vehicle (UAV) control tasks. Yin and Zhang (2014) measured the MWL variations using such features as the power of different frequency bands of multi-channel EEG signals, heartbeat interval, Heart Rate (HR), and Heart Rate Variability (HRV). Bindewald (Bindewald et al., 2014) developed an Adaptive Automation (AA) system that is able to adaptively allocate tasks between operator and automated system. Yildiz et al. (2009) used the Shannon entropy of the spectral power in different frequency bands of the EEG signals as the physiological indicators of operators' alertness level. Noel et al. (2005) adopted artificial neural networks to classify MWL. Ke et al. (2014) developed cross-task performance-based feature selection and regression model for the MWL. Lin and Zhang (Lin et al., 2013) combined kernel fisher discriminant analysis and kernel principal component analysis for mental workload recognition. In this paper, we proposed a new framework for MWL classification.

The physiological signal is weak in amplitude, highly non-linear and statistically non-stationary and noisy, thus it is difficult to recognize different awareness status from non-stationary physiological signals. Feature extraction is the key. Some commonly-used feature extraction methods include Power Spectrum (Pfurtscheller et al., 2016), Fast Fourier Transform (FFT; Varsta et al., 2000), Auto-Regressive (AR) model (Burke et al., 2005), Independent Component Analysis (ICA; Zhou and Gotman, 2009), Bispectrum estimation (Zhang et al., 2000), and neural networks (Anderson et al., 1995). When estimating the power spectral density of a short EEG time-series signal, the statistical characterization is insufficient. FFT has limitation in analyzing the spectrum of time-varying signals. AR model can describe the time-varying characteristics of the signal, but it is more suitable for analyzing stationary signals. In this paper, we used Short-Time Fourier Transform (STFT; Tsai et al., 2015) to extract the time-frequency features at each time instant from the measured time-series signals.

The rest of this paper is organized in the following way. In Section Data Acquisition Experiments, we described the experimental design paradigm and procedure for physiological data acquisition. Furthermore, STFT is employed to extract the physiological features (markers/measures) of the MWL. In Section Methods, we compared five different CNN model structures and four different parameter optimization methods. Subsequently we compared three different CNN ensemble (fusion/aggregation) approaches and discussed the model selection issue in selective ensemble learning in Section MWL Classification Results. Finally, Section Discussion draws some meaningful conclusions from the comprehensive comparative results presented previously.

## Data acquisition experiments

### Participants

**Figure 1:**
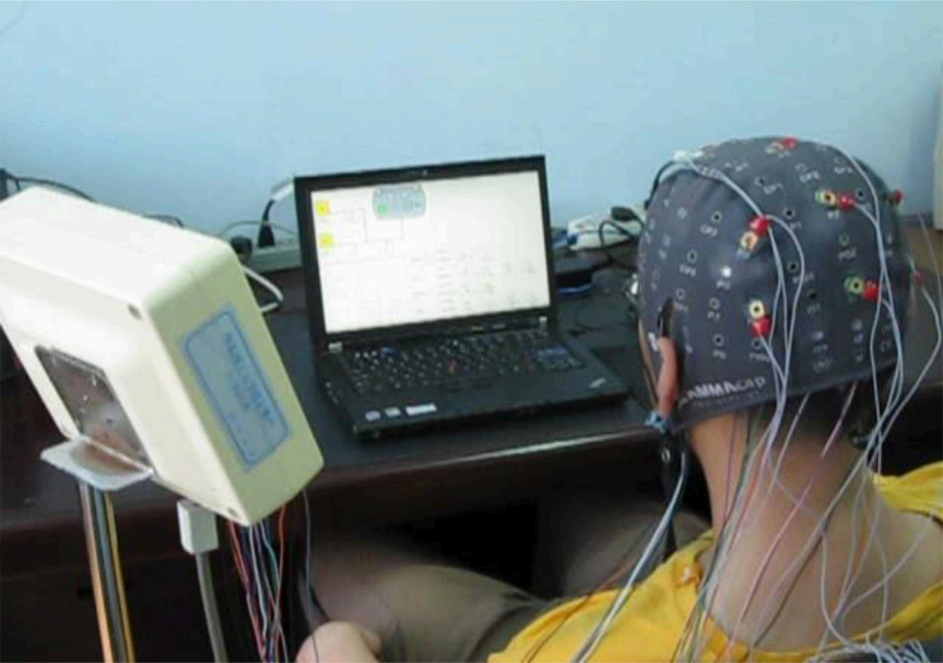
Data acquisition experimental setup.

Six volunteers participated in the experiment. All participants (coded by A - F; 22–24 y/o, all male) were graduate students recruited from East China University of Science and Technology, Shanghai, China. Each participant was informed by the experimenter of the purpose and procedure of the experiments before the formal experimental sessions. Then he was trained on the execution of the aCAMS manual control tasks for about 12 h to make him get familiar to the aCAMS task environment and manual control tasks. The training session was scheduled not too long to reduce the possible performance distortion caused by the so-called Learning Effect (LE). The participants were informed to ensure 8 h of sleeping and to prohibit smoking, drinking, or any other caffeine-contained beverages before the experiment. The experimental setup is shown in Figure 1.

### Experimental environment

In order to reliably examine the change of operator MWL levels under different levels of task-load, the laboratory where the experiments are carried out must be sound-proof. The experimental data acquisition need to meet the following three fundamental criteria: (1) The simulation platform should be complex enough to simulate the real-world operational environment with certain level of task fidelity in the lab setting, with an aim to analyze/assess quantitatively the operator performance; (2) the experiment must be able to achieve real-time monitoring the operator MWL state; and (3) the experiment must involve different levels of task difficulty.

In view of the above criteria, we used the simulation software, Automation-enhanced Cabin Air Management System (aCAMS) that was redeveloped by the FGAIO group, Technical University of Berlin, Germany. The operator was required to regulate the air quality near its optimal level, which is measured by a set of output variables of several subsystems. Two parameters, i.e., the number of subsystems (NOS) manually controlled by the operator and the binary (standard vs. high) level of actuator sensitivity (AS), were used to label the target (or true) MWL level of each data point in high-dimensional feature space at each time step. The target MWL levels will be used to evaluate the classification performance subsequently. When AS level is HIGH, it is more difficult for the operator to simultaneously regulate the output of several subsystems to the respective target zones. The different combinations of NOS and AS parameters were designed to generate different levels of MWL in the experimental paradigm.

It is noted that the basic goal of this work is to classify the operator MWL levels using physiological data. Thus, experiments were conducted by using the Nihon Kohden® signal measurement system to collect physiological signals (EEG, EOG, and ECG) of the operator executing process control tasks in collaboration with the aCAMS.

### Experimental tasks

The aCAMS consists of four subsystems, namely Oxygen (O2) concentration, Pressure (P), Carbon dioxide (CO2) concentration, and Temperature (T). The four subsystems work together to sustain air quality in the closed cabin of a manned spacecraft or deep-sea submarine for example. Each subsystem has two control modes, i.e., manual operational mode by operator and automatic mode by the computer-based aCAMS, respectively. The operator's task is to control the outputs of several or all (4) subsystems in their preset target zones (O2: 19.6–20.0%; P: 990–1025 mbar; CO2: 0.2–0.6%; T: 19.5–22.0°C).

### Experimental procedure

The major experimental tools included aCAMS simulation software and physiological signal measurement system. The experimenter monitored the real-time signal acquisition process to detect if there are unexpected events (e.g., bad contact of the electrodes with the scalp) or abnormalities in the recorded signals.

**Figure 2:**
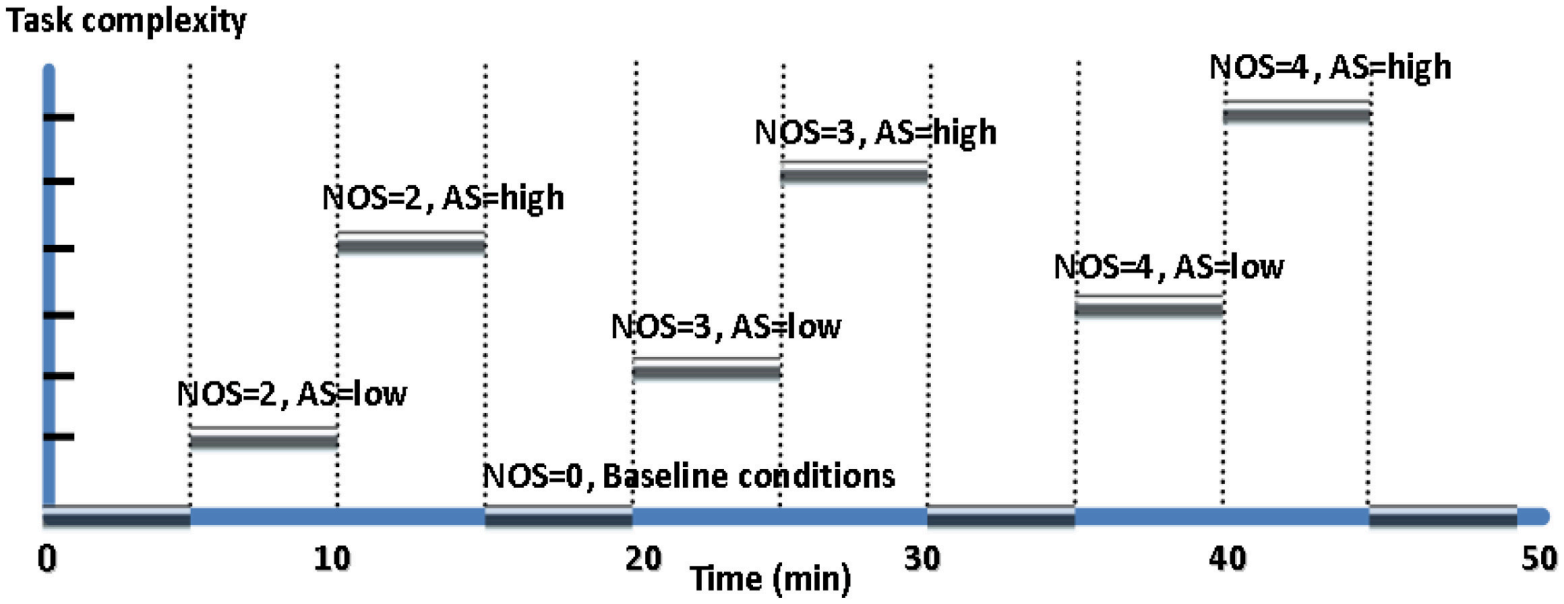
The 10 task-load conditions in a session of experiment.

**Figure 3:**
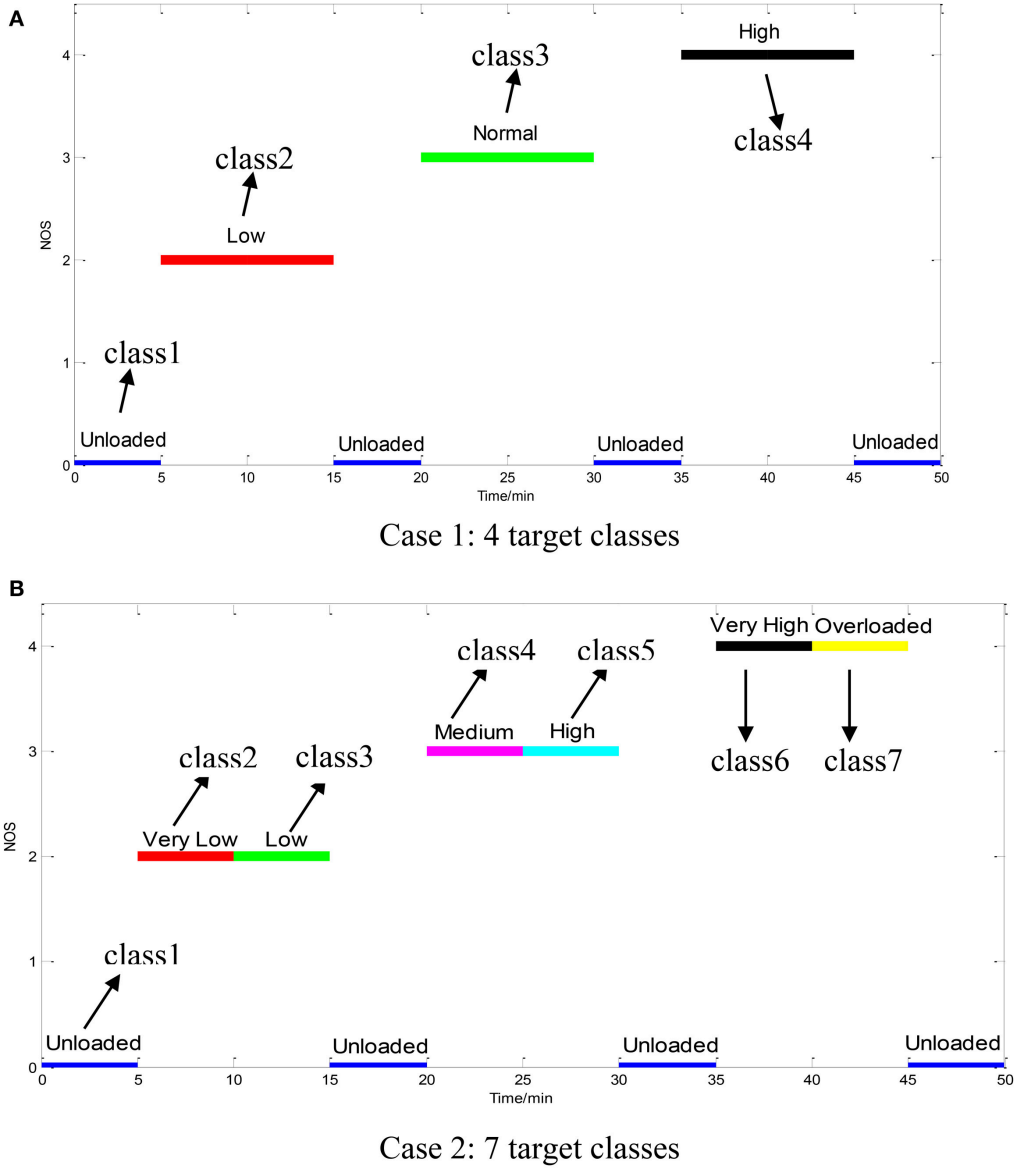
The determination of the target classes in an experimental session: **(A)** Case 1: 4 target classes; **(B)** Case 2: 7 target classes.

The task-load conditions in a session are shown in Figure 2 and Table 1. Each experimental session was conducted between 2 and 5 p.m. For each participant, two experimental sessions with the same design and procedure were performed. The 12 sessions (= 6 participants × 2 sessions per participant) were coded simply by A1, A2; B1, B2;…; F1, F2, respectively. As shown in Figure 3, each session comprises 10 task-load conditions, each of which lasted for 5 min. The condition #1, 4, 7, and 10 are baseline conditions (corresponding to unloaded resting state of the operator), during which all the subsystems were automatically controlled by the aCAMS software (i.e., NOS = 0). In condition #2 and 3, the participant was asked to manually control two subsystems that may malfunction with automatic controllers (i.e., NOS = 2). In condition #5 and 6, the participant was asked to manually control three possibly faulty subsystems (i.e., NOS = 3). In condition #8 and 9, the participant was required to manually control all four subsystems (i.e., NOS = 4). It is noted that the AS parameter is also different in different conditions, i.e., the AS was low in condition #2, 5, and 8, but high in condition #3, 6, and 9. During each session of experiment, the data sampling rate is 500 Hz.

**Table 1:** Task-load conditions in an experimental session.

**Task-load condition**	**Mode of control**	**NOS**	**AS**
1	Auto	–	–
2	Manual/Auto	2 (O_2_/P)	LOW
3	Manual/Auto	2 (O_2_/ P)	HIGH
4	Auto	–	–
5	Manual/Auto	3 (O_2_/P/CO_2_)	LOW
6	Manual/Auto	3 (O_2_/P/CO_2_)	HIGH
7	Auto	–	–
8	Manual	4 (O_2_/P /CO_2_/T)	LOW
9	Manual	4 (O_2_/P/ CO_2_/T)	HIGH
10	Auto	–	–

### Data preprocessing

**Figure 4:**
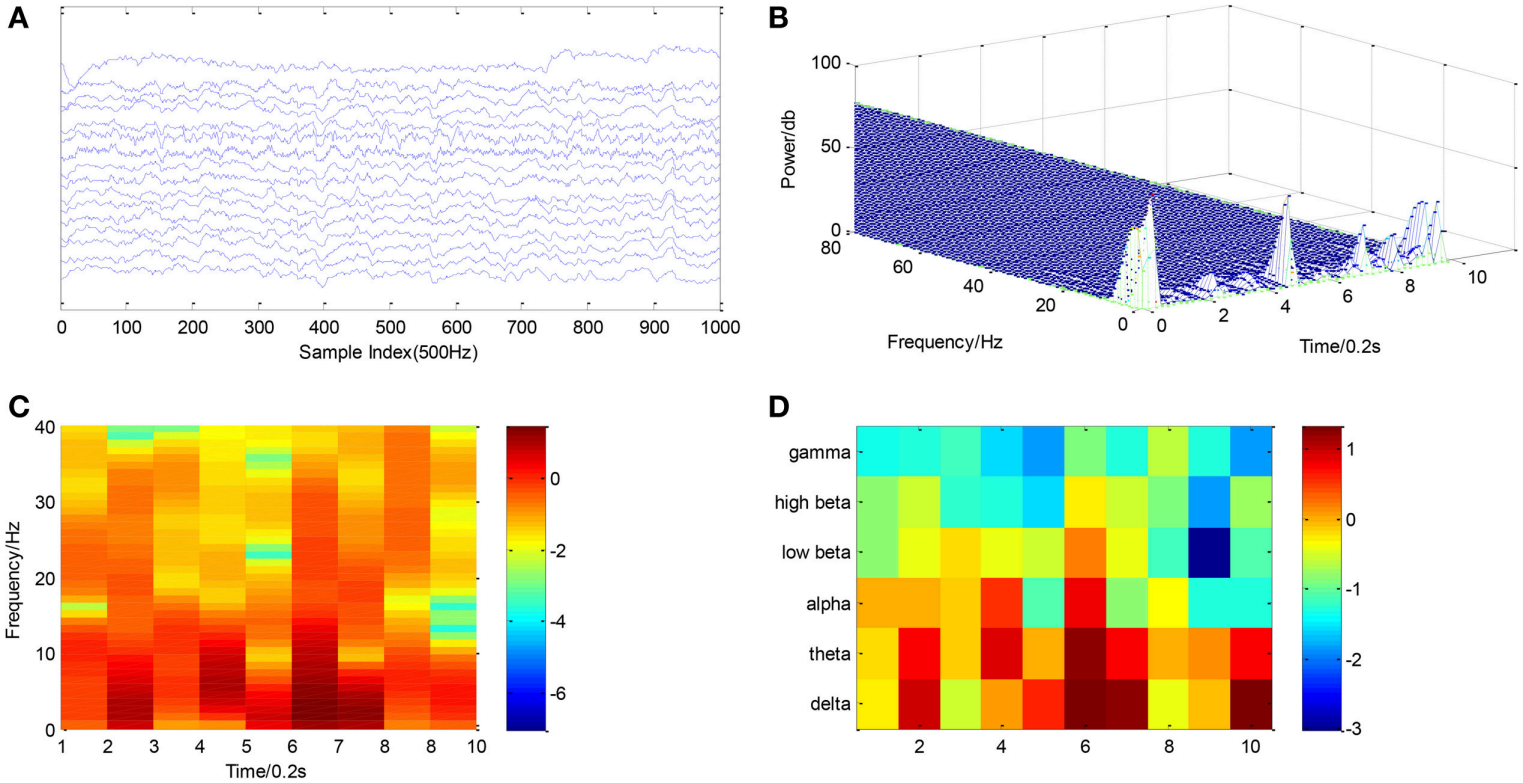
Processing of a 2-s. segment of physiological data: **(A)** Raw multi-channel EEG signals; **(B)** 3D power spectrum; **(C)** The STFT-extracted features; **(D)** The average feature in each frequency band.

The measured raw data were firstly filtered by using a low-pass (0–40 Hz) filter to reduce the higher-frequency noise. The next important step is to extract the MWL features. The FFT is suitable for statistically stationary signals, unfortunately the physiological signals are usually non-stationary in nature. Hence STFT will be adopted to extract the time-frequency features. An example of the STFT analysis results is shown in Figure 4.

Six EEG rhythm power features are most widely used, viz. delta (1–4 Hz), theta (5–8 Hz), alpha (9–13 Hz), lower beta (14–16 Hz), higher beta (17–30 Hz), and gamma (31–40 Hz) (Mirowski et al., 2009). The six frequency bands have a significant correlation with mental workload.

The STFT of a signal *x*(*t*) is given by:

(1) $$\begin{array}{r} STFT\left(m,\omega \right)=\underset{m}{\sum }x\left[m\right]g\left[m-\tau \right]e^{-jmw} \end{array}$$

Where *g*(·) is a window function used to segment the signal. The Fourier transform of the signal at different times can be obtained by constantly sliding the window. Given, *g*(·), *e*^*iw*^ function and frequency limit, the STFT of the signal can be computed by Equation (1).

After applying the STFT, the average energy of each frequency band is used as the EEG feature. In each task-load condition, the first and last 5-s. data segments were removed from the final dataset in order to guarantee high quality of the data, as a result, the EEG signal in each channel consists of 1450 data points with a sampling interval of 2 s.

Task performance data, Time In Range (TIR), can be used to determine the target labels of MWL at each time instant as well as to evaluate the overall performance of the HM system. *TIR* is defined as the time ratio of the aCAMS system in target zones in a certain period of time:

(2) $$\begin{array}{r} TIR=\frac{r_{O_{2}}\left(k\right)+r_{P}\left(k\right)+r_{CO_{2}}\left(k\right)+r_{T}\left(k\right)}{4} \end{array}$$

Where *r*_*O*_2__(*k*), *r*_*P*_(*k*), *r*_*CO*_2__(*k*), and *r*_*T*_(*k*) and are the Boolean variable for the respective subsystem. if the subsystem is in target zone at time instant *k*, otherwise it takes the value of zero.

**Figure 5:**
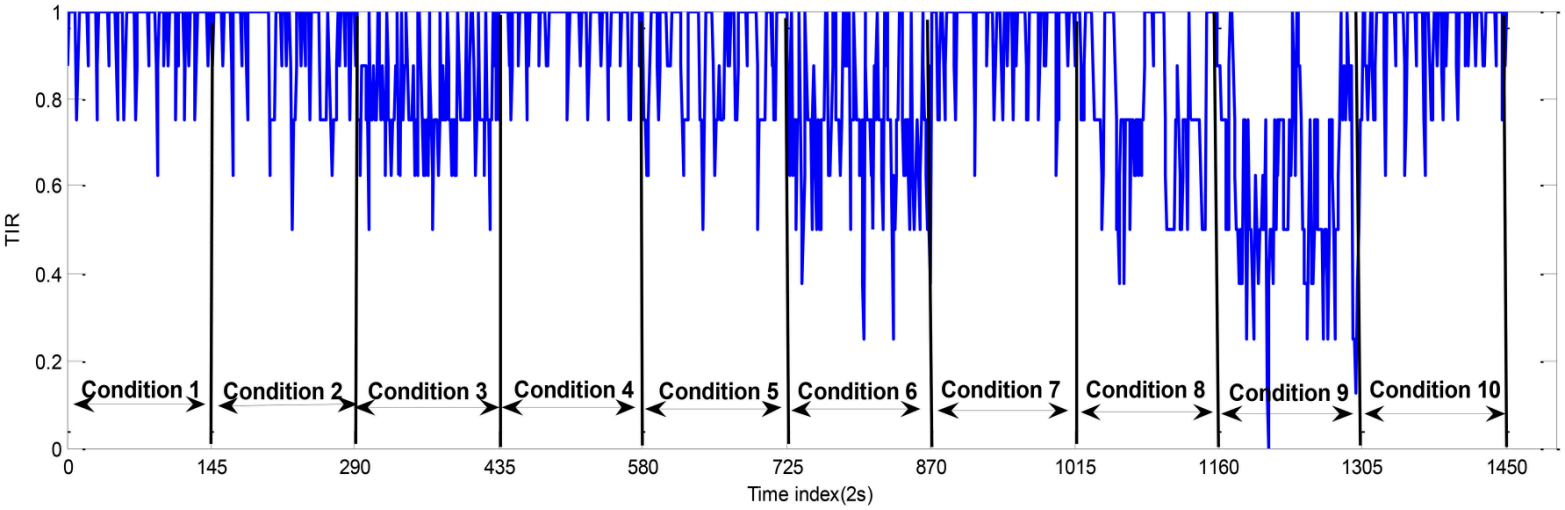
The task performance (TIR) data (participant A; session 1).

The average TIR value in the unit interval [0, 1] for each loaded condition is presented in Table 2. The TIR data measured from participant A are shown in Figure 5. The smaller the TIR is, the lower performance the operator has. Table 2 shows the TIR for each participant with three levels of NOS (2, 3, and 4). NOS can take the value of 2 (condition #2 and #3), 3 (condition #5 and #6), or 4 (condition #7 and #8). It can be seen that the average TIR is much higher in condition #1 (NOS = 2) than condition #2 (NOS = 3) and #3 (NOS = 4). The change of TIR is caused by the fluctuation of MWL levels. We used one-way ANOVA technique to compare the operator performance across the three task-load conditions and the results show that the TIR is significantly different under different conditions [*F*_(2, 69)_ = 9.25, *p* < 0.05]. The condition #4 is unloaded as there is no manual task imposed on the operator. In this way, the MWL can be classified into four classes (Unloaded, Low, Normal, and High) based on the discrete NOS variable in Case 1.

**Table 2:** Task performance (*TIR*) data.

**Session**	**NOS = 2**		**NOS = 3**		**NOS = 4**	
	**Cond. 2**	**Cond. 3**	**Cond. 5**	**Cond. 6**	**Cond. 8**	**Cond. 9**
A1	0.93	0.79	0.90	0.74	0.80	0.60
A2	0.96	0.72	0.96	0.68	0.77	0.42
B1	0.95	0.76	0.90	0.66	0.81	0.45
B2	0.93	0.79	0.91	0.71	0.80	0.40
C1	0.97	0.76	0.93	0.69	0.84	0.55
C2	0.96	0.79	0.97	0.73	0.89	0.52
D1	0.94	0.75	0.90	0.66	0.82	0.52
D2	0.97	0.74	0.90	0.70	0.83	0.57
E1	0.98	0.87	1.00	0.84	0.96	0.72
E2	0.97	0.86	1.00	0.84	0.94	0.73
F1	0.98	0.86	0.99	0.78	0.94	0.70
F2	0.96	0.86	0.99	0.76	0.97	0.68
Average	0.959	0.795	0.945	0.732	0.863	0.571

It is noted that another binary (or Boolean) variable AS takes the value of LOW or HIGH, hence there are six (= 2^*^3) possible combinations of AS and NOS. The two-way ANOVA technique was used to examine whether there is significance difference in the six possible combinations. The results show that the mean TIR is significantly different across the six combinations [*F*_(2, 66)_ = 5.6, *p* < 0.05]. The condition #7 is unloaded. Therefore, the MWL can be classified into seven classes (Unloaded, Very Low, Low, Medium, High, Very High, and Overloaded) in Case 2 if taking into account both NOS and AS.

On the other hand, clustering method can be employed to analyze the performance data of the participants. A Performance Indicator (*PI*) is defined by:

(3) $$\begin{array}{r} PI=c_{1}TIR+c_{2}NOS \end{array}$$

Where *NOS* represents the level of task difficulty and *c*_1_ = 0.7, *c*_2_ = 0.3 are two weights empirically selected.

Then *k*-means clustering technique is applied on the *PI* data. The label of the cluster obtained is regarded as the target label of each physiological data. The number of target classes is determined by the following Silhouette index:

(4) $$\begin{array}{r} S\left(i\right)=\frac{b\left(i\right)-a\left(i\right)}{\max\left\{a\left(i\right),b\left(i\right)\right\}} \end{array}$$

Where *a*(*i*) represents the average Euclidean distance between the *i*-th data point and the rest of the data points in the same cluster and *b*(*i*) represents the minimum Euclidean distance between the *i*-th data point and all data points in the rest of the clusters (other clusters).

The index *S*(*i*) defined in Equation (4) measures the degree of clustering validity, that is, the larger *S*(*i*), the higher the clustering quality.

**Figure 6:**
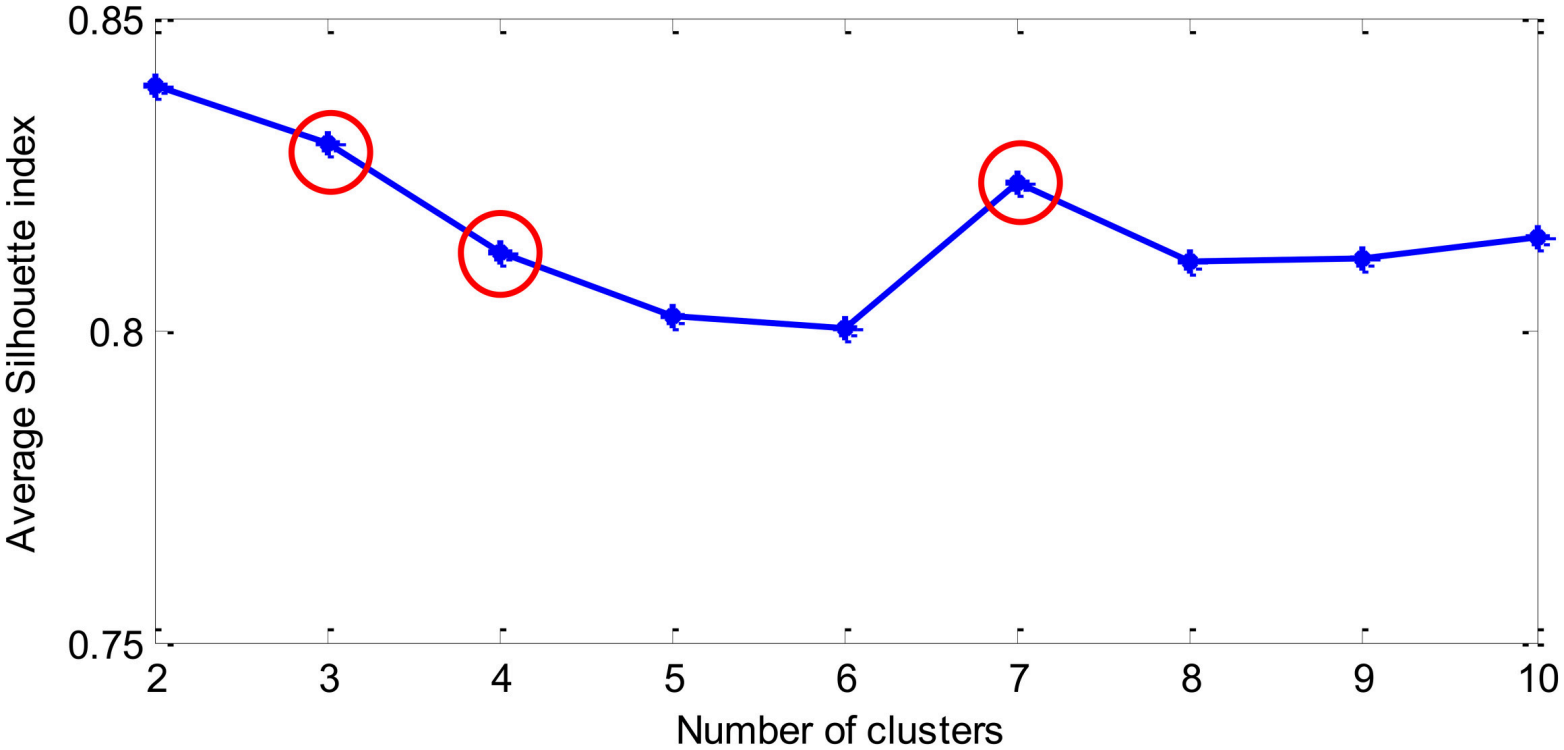
The average Silhouette index vs. the number of clusters (*K*) assumed in *K*-means clustering algorithm.

The clustering result is shown in Figure 6. It can be seen that it is appropriate to categorize the performance data into 3, 4, or 7 classes. In order to examine more subtle changes of the MWL, 4 and 7 target classes are considered here. The labels of the four target classes are Unloaded, Low, Normal, and High. For seven target classes, the labels are Unloaded, Very low, Low, Medium, High, Very High, and Overloaded.

Consequently, we may conclude that the labels generated by the classifier can represent the prediction of the true MWL levels, instead of confounded instantiations of MWL, experience and the variation in the objective task-load (the level of task difficulty or complexity).

The number of target MWL classes is determined to be either 4 or 7 based on the levels of task difficulty. The target class labels are given in Table 3. In Case 1 (4-class classification), 4 target levels of MWL can be identified based on the parameter NOS alone, i.e., unloaded level (task-load condition #1, 4, 7, and 10 with the parameter NOS = 0), under-loaded (or low) level (condition #2 and 3 with the parameter NOS = 2), normal level (condition #5 and 6 with the parameter NOS = 3), and overloaded (or high) level (the last two conditions #8 and 9 with the parameter NOS = 4). Furthermore, we consider both parameter NOS and AS to formulate a 7-class classification problem in Case 2, in which the condition #1, 4, 7, and 10 were in Unloaded level (Class 1), #2 in level Very Low (NOS = 2 and AS = LOW), #3 in level Low (NOS = 2 and AS = HIGH), #5 in level Medium (NOS = 3 and AS = LOW), #6 in level High (NOS = 3 and AS = HIGH), #8 in level Very High (NOS = 4 and AS = LOW), and condition #9 in level Overloaded (NOS = 4 and AS = HIGH). The datasets are described in terms of Name, Size, Feature Dimensionality (#D), Numbers of target classes (#C) and the size of each class in Table 3.

**Table 3:** Description of MWL datasets.

**Case**	**Size**	**No. F**	**No. C**	**Target class label**	**Size of each class**
1	2900	17	4	Unloaded/Low/Normal/High	1160/580/580/580
2	2900	17	7	Unloaded/Very Low/Low /Medium/High/Very High/Overloaded	1160/290/290/290/290/290/290

## Methods

### CNN structure

The bio-inspired CNN is a type of feedforward neural network which can be used to realize both feature extraction and classification. A basic CNN is composed of alternating convolutional and pooling layers followed by a single or multiple fully-connected layers plus a classifier layer. The CNN structure was originally applied to the problems of object recognition (LeCun et al., 2004) and handwritten character recognition (Simard et al., 2003). Hinton's group won the first prize in ImageNet competition by using Deep Convolutional Neural Network (DCNN) in 2012 (Krizhevsky et al., 2012). In 2014, Google designed an extremely DCNN, called GoogLeNet, which improved the utilization of the computing resources in the network. Szegedy et al. (2015) showed distinct advantage of the DCNN for speech recognition problem.

**Figure 7:**
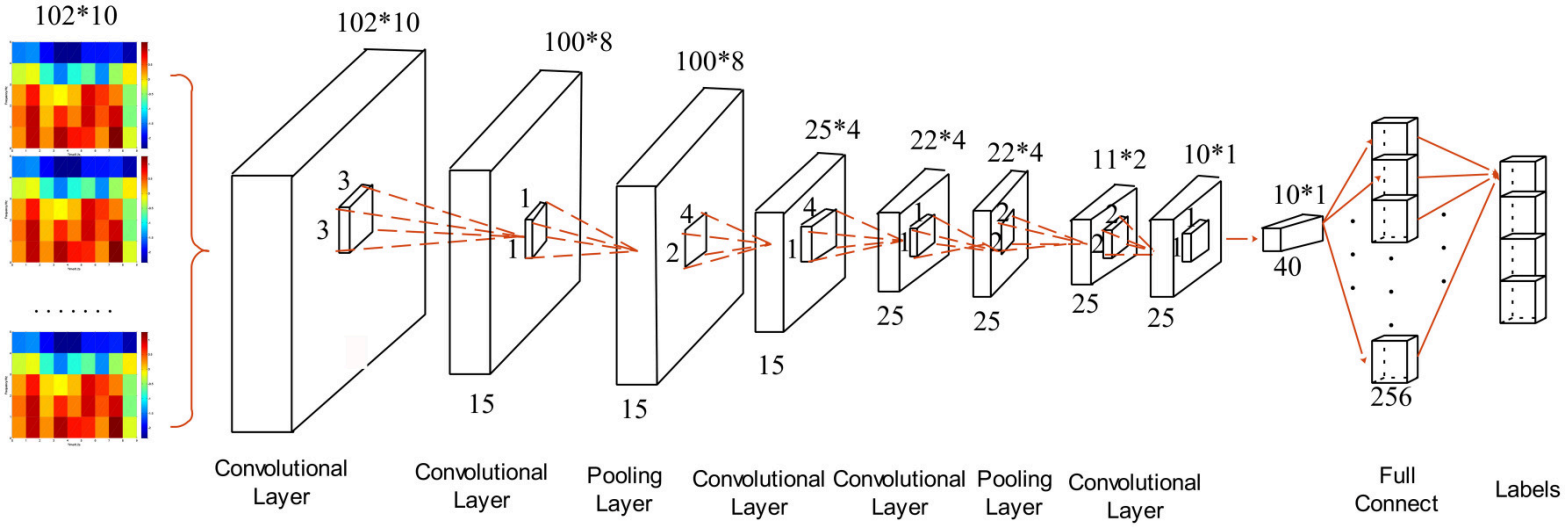
Structure of single CNN used for MWL classification.

In order to find out the optimal structure of CNN for MWL classification problem, we compared five candidate structures. The concrete parameters of individual CNNs are provided in Table 4. The parameters in convolutional layer are denoted by “Cov (size of receptive field)-(feature dimensionality).” The input matrix of the CNN has the dimension of 102 × 10. The five structures have different depth with an aim to find the best depth for the specific MWL classification problem under study. We used small (e.g., 1 × 1 or 3 × 3) convolutional filters to capture the features, e.g., CNN3 used 1 × 1 convolutional filters which can be considered as a linear transformation of the input data. The structure of CNN3 is depicted in Figure 7. The convolutional stride is 1 in all CNNs. After the first convolutional layer, we added the second convolutional layer, followed by an average-pooling layer. This is useful in enhancing the capacity of feature extraction. The alternating convolutional and pooling layers are followed by the Fully-Connected (FC) layers. The final layer is a soft-max layer. The FC layers and soft-max layer are set to be identical in all five structures. All convolutional layers used Rectified Linear Units (ReLu), rather than sigmoidal ones, to obtain improved performance.

**Table 4:** CNN structural parameters.

**Model**	**No. of layers**	**Cov.layer**	**Pool.layer**	**Cov.layer**	**Pool.layer**	**Cov.layer**	**FC**
CNN1	2	Cov3 × 3–15	Pool 4 × 2				256
CNN2	4	Cov3 × 3–15	Pool 4 × 2	Cov4 × 1–25	Pool 2 × 2	Cov4 × 1–40	256
CNN3	7	Cov3 × 3–15 Cov1 × 1–15	Pool 4 × 2	Cov4 × 1–25 Cov1 × 1–25	Pool 2 × 2	Cov2 × 2–40 Cov1 × 1–40	256
CNN4	7	Cov3 × 3–15 Cov3 × 3–15	Pool 2 × 2	Cov4 × 1–25 Cov5 × 1–25	Pool 2 × 1	Cov2 × 2–40 Cov2 × 2–40	256
CNN5	10	Cov3 × 3–15 Cov3 × 3–15 Cov3 × 3–15	Pool 4 × 2	Cov4 × 1–25 Cov4 × 1–25 Cov4 × 1–25	Pool 3 × 1	Cov2 × 1–40 Cov2 × 1–40 Cov2 × 1–40	256

### Parameter optimization algorithms

As far as the training of a deep CNN is concerned, we compared four training algorithms for the above-mentioned five CNN structures, including Nesterov Momentum (NM), Adagrad (Bengio et al., 2013), Adadelta (Khan et al., 2016) and Adaptive Moment Estimation (Adam; Kingma and Ba, 2015). We define cross entropy error as the loss function. The cross entropy represents the dissimilarity between the output and the known actual (or target) labels.

NM improves traditional momentum algorithm by using a momentum term to update the parameters. There are two adjustable parameters: one is learning rate and another is moment. We set them to be 0.0001 and 0.9, respectively. Adagrad makes a 2nd-order correction to the predictor using the previous loss functions. It can assign different learning rates for each parameter. We initialized learning rate to be 0.0001. Adadelta is an extension of Adagrad based on 1st-order information. Compared with the Stochastic Gradient Descent (SGD) or other optimization algorithms, its computational overhead is trivial. It is a robust learning method that has been applied in many situations (Kingma and Ba, 2015). Adam is designed to optimize stochastic loss function and adaptively update weights based on lower-order moments. There are three adjustable parameters: learning rate and two decay rates. We set the learning rate as 0.0001 and the two decay rates as 0.9 and 0.999, respectively.

### Ensemble CNNs

**Figure 8:**
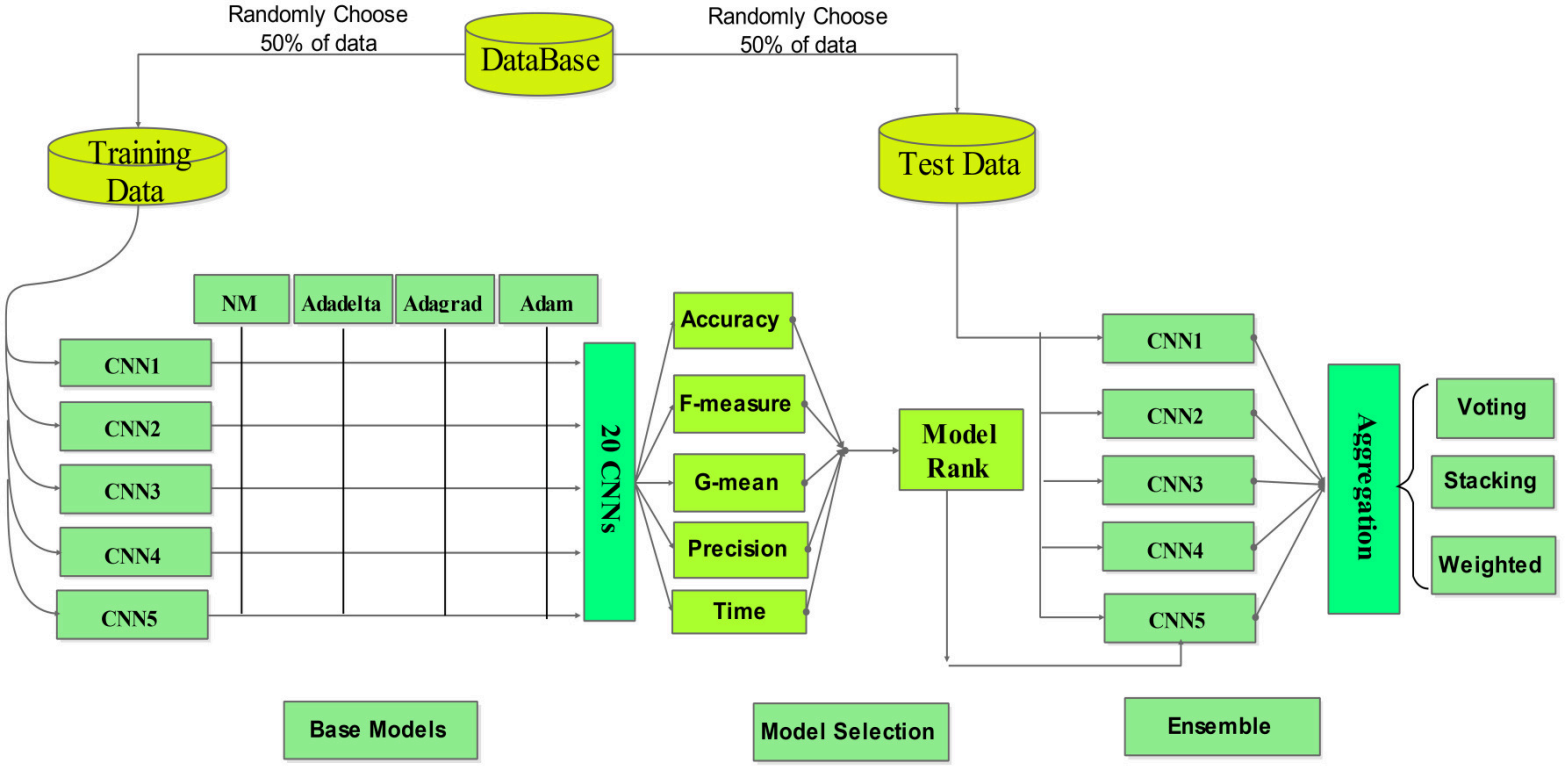
The proposed MWL classification framework.

ECNN aggregates multiple CNNs to achieve better results than using any of the base models alone (Ozcift and Gulten, 2011). In general the performance of the models to be aggregated (or combined) needs to be adequately diverse. The ensemble learning method can overcome the over-fitting issue of a single base learner. The ECNN framework is depicted in Figure 8.

Researchers have developed many ensemble learning algorithms in recent years, such as Bagging, Boosting, and Random Forest. Generally, there are two important problems in constructing an ensemble model: one is the design of base classifiers and the other is the selection of a proper aggregation approach.

The base classifiers should be sufficiently diverse. Different base classifiers can be created by using different training sets, input features, parameters, and/or learning algorithms (Padilha et al., 2016). Here we use different training sets and parameters to design base (or member) CNNs.

For the MWL classification problem of our interest, three ensemble methods, namely voting, weighted averaging and stacking, are compared for the ensemble of multiple base learners. To obtain the final classification result, the outputs of the individual models are combined by means of weighting or voting.

#### Voting

The output class label is determined through majority voting among base classifiers.

#### Weighted averaging

Let y_*j*_(*i*), *j* = 1, 2,., K represent the *i*-th output of *j*-th base model in the ensemble, the output of the ensemble classifier can be computed by weighted averaging (i.e., assign different weights to different base models and the base model with higher classification accuracy would be more weighted):

(5) $$\begin{array}{r} S\prime \left(i\right)=\overset{K}{\underset{j=1}{\sum }}\alpha_{j}y_{j}\left(i\right) \end{array}$$

Where the weights α_*j*_ can be chosen based on the ranking of accuracy of the individual models:

(6) $$\begin{array}{r} \alpha_{j}=\frac{R\left(A_{j}\right)}{\overset{K}{\underset{i=1}{\sum }}R\left(A_{k}\right)} \end{array}$$

In Equation (6), *R*(·) denotes the ranking index of the model accuracy, e.g., *R*(·) = *K* if the model has the highest accuracy while *R*(·) = 1 if the model has the lowest accuracy.

#### Stacking

A typical stacking structure involves two layers. The 1st layer contains a number of base classifiers. The outputs of these base classifiers are combined by a meta-classifier to obtain the final classification result in the 2nd layer. The training procedure of the stacking can be divided into the following two steps:

**Step 1:** Train each base classifier in the first layer by 5-fold cross-validation. The outputs of base classifiers are used as the training data in the 2nd layer. For the training set {(*x*_1_, *y*_1_), …, (*x*_*n*_, *y*_*n*_)} and K base classifiers, the training set are divided into five parts. Each part is used as the testing set and the rest as the training set. For each sample *x*_*i*_, we get K different outputs $\left(y_{i}^{1},\ldots ,y_{i}^{K}\right)$ from the K base classifier models.

**Step 2:** The samples $\left\{<\left(y_{i}^{1}\ldots y_{i}^{k}\right),y_{i}>\right\}$ are used to train the classifier in the 2nd layer. After training, the output of the meta-classifier is the predicted class label.

All the data analysis programs were written by using python 2.7 and MATLAB 2014a and run on a PC with a single Intel core i5 CPU, 4-GB memory, and Window seven operating system.

## MWL classification results

In this section, we will compare the performance of five CNN models in the two classification cases, Case 1 and 2. For the purpose of classification performance evaluation and comparison, we first concatenate (combine) the data from the first and second session per participant. Then we randomly extract 50% of the dataset as the training data and the rest as the testing data.

### Performance metrics

Accuracy defined as the percentage of the correctly classified data in the test set:

(7) $$\begin{array}{r} Accuracy=\frac{tr}{N} \end{array}$$

Precision defined by:

(8) $$\begin{array}{r} Precision=\frac{1}{C}\overset{c}{\underset{i=1}{\sum }}\frac{tr_{i}}{tp_{i}} \end{array}$$

F-measure defined by:

(9) $$\begin{array}{r} F=\frac{2}{7}\overset{c}{\underset{i=1}{\sum }}\frac{Precision_{i}*Recall_{i}}{Precision_{i}+Recall_{i}} \end{array}$$

G-mean defined by:

(10) $$\begin{array}{r} Gmean={\left(\overset{c}{\underset{i=1}{\prod }}\frac{tr_{i}}{n_{i}}\right)}^{\frac{1}{c}} \end{array}$$

Where *c* represents the number of target classes, *N* is the number of data points, *n*_*i*_ is the number of data in the *i*-th class, *tr*_*i*_ is the number of the correctly classified data, is the number of data correctly classified as the *i*-th class, and is the *tp*_*i*_ total number of data classified as the *i*-th class.

### Optimal network structure and parameter optimization algorithm

In this section, we will examine the best depth of a CNN and the most appropriate parameter optimization algorithm for the MWL classification problem. Furthermore, we will compare the proposed method with existing mainstream methods.

The dataset is evenly divided into a training set and a testing set of equal size (50% of the original dataset) at random. The same data analysis experiment was repeated for five runs. The numbers of convolution filters in the 1st, 2nd, and 3rd layer are set to be 24, 12, and 10, respectively. The stride is 1 in each layer. We used a small number of convolution layers due to the small size of the training dataset. According to Roth et al. (2015), this parameter setting can enhance the performance of each CNN model.

Table 5 gives the classification confusion matrix in Case 1 and 2 for Participant A. It can be seen that the unloaded class and high class have much higher classification accuracy than low and normal class. From the last row of Table 5, in Case 1 it achieved a testing classification accuracy of 91.2% for unloaded class, which is 4.3, 6.1, and 3.0% higher than Low, Normal, and High class, respectively. The Low and Normal classes have the most misclassified data probably because they are more similar. In general, the average classification results in Case 2 are worse than in Case 1. The number of misclassified data in unloaded, Very High and Overloaded class is less than other classes. The Low and Medium class have the most misclassified data. There is a similar result in Case 1.

Table 5The MWL classification confusion matrix of NM-CNN3 (participant A).

**Table:** 

**Case 1**				
**Estimated class**	**Target class**			
	**Unloaded**	**Low**	**Normal**	**High**
Unloaded	585	10	7	2
Low	7	264	12	1
Normal	8	7	260	13
High	1	3	6	264
*ACCclass*	97.3	93.0	91.2	94.3

**Table:** 

**Case 2**							
**Estimated class**	**Target class**						
	**Unloaded**	**Very Low**	**Low**	**Medium**	**High**	**Very High**	**Overloaded**
Unloaded	588	1	4	4	1	1	1
Very Low	4	115	3	2	0	2	2
Low	3	9	132	2	3	1	0
Medium	7	4	8	116	5	1	4
High	1	0	1	6	138	3	0
Very High	0	0	6	2	5	128	1
Overloaded	1	1	0	4	0	0	130
*ACCclass*	97.4	88.5	85.7	85.3	90.8	94.1	94.2

Twenty base classifiers are formed by combining four optimization methods and five CNN models. The data analysis results are shown in Tables 6, 7. The best models are selected by considering four indices: accuracy, precision, F-measure, G-mean and require training time. From column 3–6 in Table 6, we can find that NM-CNN3 leads to the best Precision and F-measure index, but Adagrad-CNN4 has the best Accuracy and G-mean index. In terms of computational efficiency, NM-CNN3 takes much less training time, almost one-third of Adagrad-CNN4. Therefore, NM-CNN3 was selected as the best model in Case 1. The ranking of the 20 models are shown in the last column of Table 6. Similarly, from Table 7 we can find that NM-CNN3 has three best indices: accuracy of 89.6%, precision of 88.6 and F-measure of 90.3%. Moreover, the training time required is 661 s (about 10 min), which is acceptable. It can be found that NM-CNN3 achieved the best performance in both cases. i.e., 88.6% in Case 1 (82.1% in Case 2) in terms of average precision, and that Adagrad-CNN1 resulted in the worst performance in both cases, 18.39–44.21% (22.71–40.59%) lower than NM-CNN3.

**Table 6:** Comparison of average accuracy (%), Precision (%), F-measure (%), G-mean (%), and required training time (sec.) of 5 CNN models optimized by 4 optimization algorithms in Case 1.

**Model**	**Opt. algorithm methods**	**Accuracy**	**Precision**	**F-measure**	**G-mean**	**Time (s) Time(sec)**	**Rank**
CNN1	NM	78.8 ± 7.90	69.2 ± 6.01	78.5 ± 6.75	79.7 ± 08.1	201	15
	ADADELTA	77.2 ± 9.00	67.8 ± 8.67	77.1 ± 7.12	78.0 ± 9.37	243	16
	ADGRAD	66.7 ± 8.00	57.3 ± 6.90	68.7 ± 5.82	67.0 ± 8.70	232	20
	ADAM	73.0 ± 8.60	63.8 ± 5.90	72.9 ± 8.55	73.7 ± 7.12	241	18
CNN2	NM	87.9 ± 5.10	81.0 ± 7.22	86.8 ± 5.12	88.6 ± 4.91	342	6
	ADADELTA	89.1 ± 4.00	82.1 ± 5.62	88.1 ± 4.07	89.8 ± 3.93	351	3
	ADGRAD	79.0 ± 8.70	69.8 ± 9.51	78.7 ± 7.60	79.9 ± 8.82	345	14
	ADAM	86.3 ± 8.10	79.3 ± 9.92	85.3 ± 7.92	87.0 ± 7.13	354	9
CNN3	NM	89.8 ± 4.11	**88.6** ± **6.01**	**90.3** ± **4.22**	83.4 ± 4.01	721	1
	ADADELTA	88.7 ± 6.32	87.6 ± 7.73	89.3 ± 6.44	82.4 ± 6.14	743	4
	ADGRAD	81.0 ± 1.12	80.5 ± 1.12	81.7 ± 9.65	73.0 ± 1.14	723	13
	ADAM	87.8 ± 6.81	86.6 ± 2.81	88.4 ± 7.00	81.4 ± 6.62	710	7
CNN4	NM	90.1 ± 3.94	84.0 ± 5.41	88.9 ± 4.21	90.6 ± 3.81	2052	5
	ADADELTA	85.6 ± 8.22	84.3 ± 3.00	84.5 ± 7.93	86.2 ± 8.01	2045	10
	ADGRAD	**90.3** ± **4.00**	84.3 ± 5.02	89.1 ± 4.32	**90.8** ± **3.92**	2082	2
	ADAM	86.9 ± 5.55	80.1 ± 6.91	85.6 ± 5.71	87.5 ± 5.42	2021	8
CNN5	NM	83.4 ± 8.00	75.1 ± 9.63	82.7 ± 7.42	84.3 ± 7.82	2172	12
	ADADELTA	84.3 ± 0.72	76.6 ± 8.81	83.1 ± 0.72	85.0 ± 0.07	2192	11
	ADGRAD	74.8 ± 1.07	65.9 ± 1.12	74.5 ± 0.98	75.7 ± 0.11	2151	17
	ADAM	67.2 ± 1.89	61.6 ± 1.53	72.0 ± 1.07	53.9 ± 3.84	2140	19

**Table 7:** Comparison of average accuracy (%), Precision (%), F-measure (%), G-mean (%), and required training time (sec.) of 5 CNN models optimized by 4 optimization algorithms in Case 2.

**Model**	**Opt. algorithm methods**	**Accuracy**	**Precision**	**F-measure**	**G-mean**	**Time (s) Time(sec)**	**Rank**
CNN1	NM	74.6 ± 7.62	63.8 ± 6.86	75.8 ± 5.81	75.3 ± 6.23	201	17
	ADADELTA	71.6 ± 5.00	60.6 ± 4.41	73.2 ± 3.93	72.3 ± 5.51	241	18
	ADGRAD	59.4 ± 4.91	50.8 ± 3.22	65.7 ± 3.01	57.1 ± 6.72	235	19
	ADAM	64.2 ± 4.42	54.2 ± 3.21	68.2 ± 3.31	63.8 ± 5.01	242	20
CNN2	NM	87.4 ± 3.91	79.4 ± 5.32	86.3 ± 3.82	88.2 ± 3.72	322	6
	ADADELTA	85.9 ± 8.00	77.6 ± 9.31	85.3 ± 7.28	86.7 ± 8.01	321	8
	ADGRAD	79.8 ± 7.45	69.6 ± 7.87	79.5 ± 6.70	80.8 ± 7.43	323	12
	ADAM	87.7 ± 3.83	79.7 ± 5.32	86.7 ± 3.82	88.5 ± 3.61	330	5
CNN3	NM	**89.6** ± **3.71**	82.1 ± 5.37	**88.6** ± **3.71**	**90.3** ± **3.51**	661	1
	ADADELTA	89.0 ± 3.72	81.4 ± 5.00	88.0 ± 3.85	89.7 ± 3.64	674	3
	ADGRAD	80.1 ± 7.40	70.6 ± 6.21	79.8 ± 6.41	81.1 ± 7.44	682	11
	ADAM	88.3 ± 4.54	80.8 ± 6.42	87.3 ± 4.71	89.1 ± 4.35	661	4
CNN4	NM	89.5 ± 4.92	**82.5** ± **6.65**	88.5 ± 5.11	90.2 ± 4.73	1871	2
	ADADELTA	85.6 ± 7.20	74.2 ± 8.72	84.7 ± 7.17	86.4 ± 7.04	1881	9
	ADGRAD	82.9 ± 8.22	74.1 ± 9.33	82.1 ± 7.82	83.7 ± 8.10	1934	10
	ADAM	75.5 ± 1.68	68.1 ± 1.47	77.9 ± 1.10	69.3 ± 3.20	1872	16
CNN5	NM	85.7 ± 6.11	77.3 ± 7.44	84.9 ± 5.83	86.6 ± 6.02	2071	7
	ADADELTA	78.6 ± 9.63	69.1 ± 9.72	78.3 ± 8.64	79.5 ± 7.00	2045	14
	ADGRAD	75.7 ± 9.33	66.1 ± 9.21	75.5 ± 8.52	76.7 ± 9.44	2010	15
	ADAM	78.7 ± 9.45	68.9 ± 9.00	78.5 ± 8.10	79.6 ± 9.62	2003	13

In general the CNN performance improves with the increase of its depth. The CNN1 achieved poor results because the too-shallow architecture prevents the network from learning highly non-linear features. The CNN5 performed poorly as it demands more data to train and is susceptible to over-fitting phenomenon due to its complex structure. By a comparison of the four different parameter optimization algorithms, properly parameterized NM achieved better performance, whereas ADAM contains only one free parameter (i.e., learning rate).

**Figure 9:**
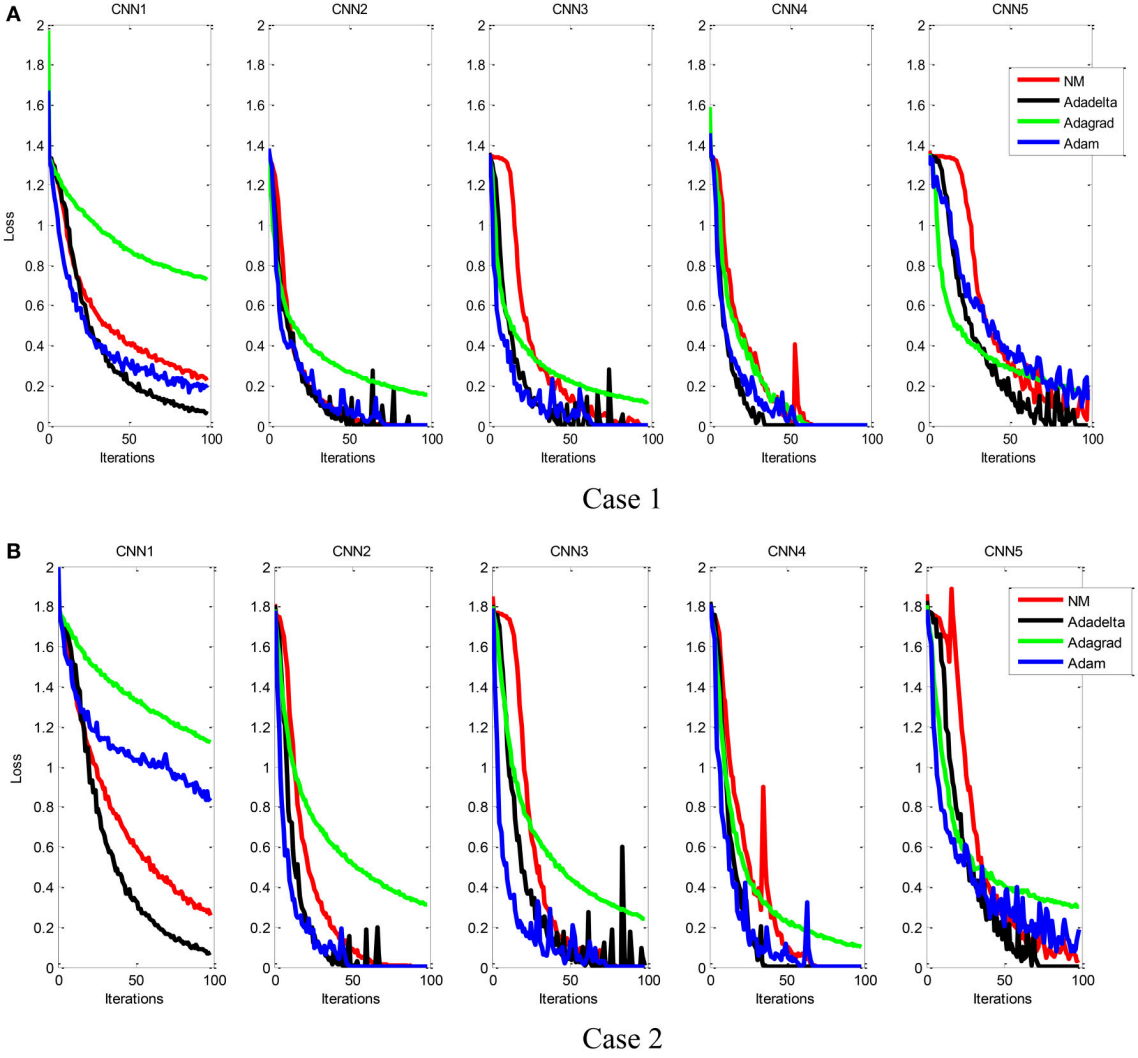
Comparison of the training convergence rate of five CNN models, each of which was trained by using four different parameter optimization algorithms. Case 1 **(A)** and Case 2 **(B)**.

Figure 9 showed the training convergence curves of five different CNN models. We can find that the Adam algorithm exhibits rapid convergence at the beginning of the training process, but unstable and slow training process later on. As shown in Table 6, the NM algorithm with properly selected parameters achieved the best performance.

**Figure 10:**
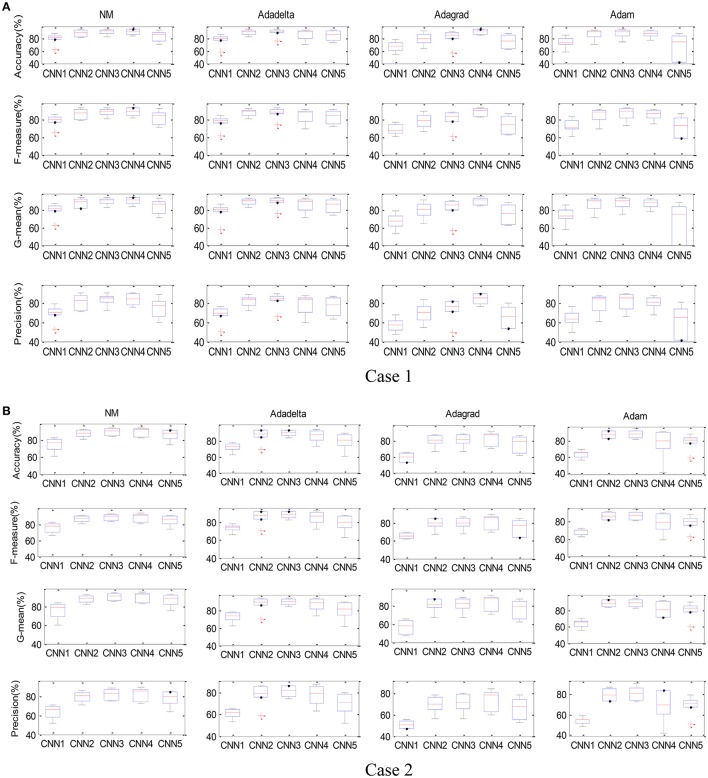
The box-whisker plot of the combinations of five CNN models and four parameter optimization algorithms. Case 1 **(A)** and Case 2 **(B)**.

The box-whisker plot of the five models and four optimization algorithms is depicted in Figure 10, showing the results for six participants in both cases. We can find that the accuracy for each combination of the model and optimization algorithm fluctuated, but there are still some outliers, e.g., the NM-CNN1, Adadelta-CNN1, Addadelta-CNN3, and Adagrad-CNN3 in Case 1 and the Adadelta-CNN2 and Adam-CNN5 in Case 2. The distribution of four performance indices, viz. Accuracy, F-measure, G-means and Precision, for the NM-CNN3 is obviously better than other 19 combinations.

**Figure 11:**
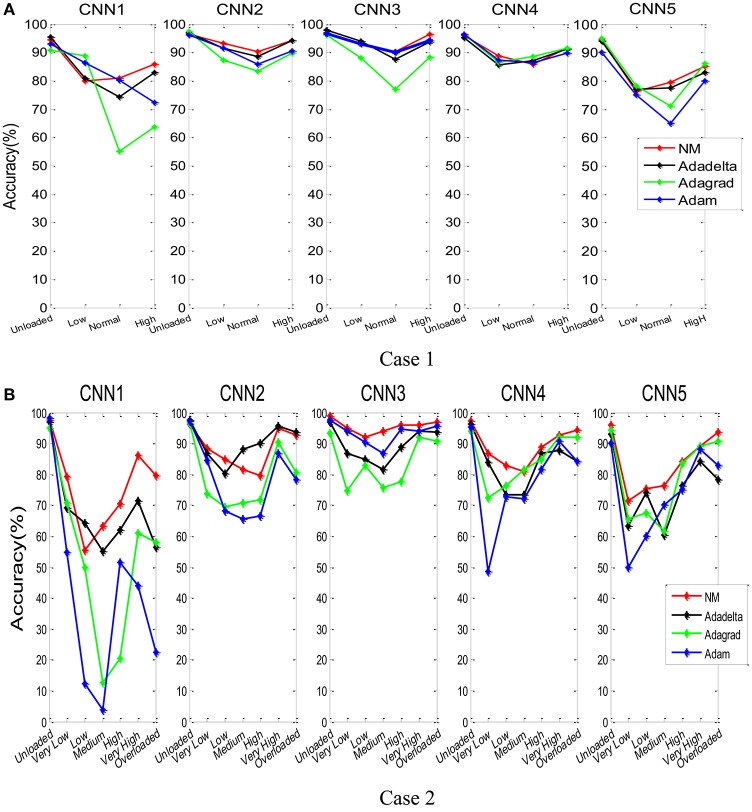
The testing accuracy for each class for the combinations of five CNN models and four parameter optimization algorithms (participant A). Case 1 **(A)** and Case 2 **(B)**.

Figure 11 showed the classification accuracy of each class for participant A, in which the horizontal axis represents the class label. In Case 1, NM-CNN3 achieved better classification performance in unloaded and high class. More data points are misclassified in Low class and Normal class, indicating that it is harder to distinguish these two similar (neighboring) classes. Non-etheless, the NM-CNN3 achieved better classification performance on these two classes. For example, it achieved a testing classification accuracy of 91.2% in Normal class, which is 3.7, 14.1, and 1.3% higher than Adadelta-CNN3, Adagrad-CNN3, and Adam-CNN3, respectively. Similarly, in Case 2 the Unloaded, Very High and Overloaded class has higher classification accuracy.

### Ensemble results

Firstly we used resampling (i.e., bootstrapping) strategy (i.e., 80% of samples in the training set were taken in each random run) to form diverse training datasets for the base CNN models. Then we designed the ensemble classifier. Since the base classifiers must be rather different from each other, different CNN structures and different parameter optimization algorithms were explored. We compared four different ensemble strategies: (1) Select the best one in each of the five CNN models for ensemble (i.e., five models from the 20 candidate models), whose results were shown as Weighted1 and Voting1 in Tables 8, 9; (2) Select the best and worst models in each of the five CNN models (i.e., 10 models from the 20 candidate models), shown as Weighted2 and Voting2 in Tables 8, 9; (3) Select the top (best) 10 models from the 20 (= 5^*^4) candidate models, shown as Weighted3 and Voting3 in Tables 8, 9; and (4) Select all the 20 candidate CNN models, shown as Weighted4 and Voting4 in Tables 8, 9. In the first strategy, we selected the NM-CNN1, Adadelta-CNN2, NM-CNN3, Adagrad-CNN4 and Adadelta-CNN5 as the five base classifier models in Case 1, whose normalized weights are set to be 0.1, 0.2, 0.4, 0.2, and 0.1, respectively. Then the outputs of the base models were aggregated by means of weighted averaging and majority voting approaches. The final results are shown in Table 8, from which we can find that the ensemble model outperforms individual base models and the weighted averaging approach achieved the best performance. Specifically, Weighted3 leads to the best classification Accuracy of 93.8%, which is 4, 7.4, 1.8, 3.6, 2.9, 3.4, 0.1, 0.3, and 1% higher than NM-CNN3, Stacking, Weighted1, Voting1, Weighted2, Voting2, Voting3, Weighted4, and Voting 4, respectively. Similarly, Weighted3 also achieves the best precision, F-measure, and G-means. The NM-CNN1, Adam-CNN2, NM-CNN3, NM-CNN4, and NM-CNN5 were selected as the base models in Case 2, whose normalized weights are assigned to be 0.1, 0.2, 0.3, 0.25, and 0.15, respectively. The ensemble results are shown in Table 9. From the 2nd column of Table 9, we can find that in Case 2 the Weighted3 approach also achieved the best average classification accuracy of 93.2%.

**Table 8:** The subject-average classification performance of different models in Case 1.

**Model**	**Accuracy**	**Precision**	**F-measure**	**G-means**	**Time(s)**
NM-CNN3	89.8	88.6	90.3	83.4	721
Stacking	86.4	77.2	85.6	87.3	1530
Weighted1	92	86.5	91.0	92.5	1821
Voting1	90.2	82.9	89.4	91.0	1803
Weighted2	90.9	84.0	90.1	91.6	6341
Voting2	90.4	83.4	89.6	91.2	6453
Weighted3	**93.8**	**89.4**	**92.9**	**94.2**	7504
Voting3	93.7	88.9	92.8	94.1	7530
Weighted4	93.5	88.7	92.6	94.0	12320
Voting4	92.8	87.4	92.0	93.4	12332

**Table 9:** The subject-average classification performance of different models in Case 2.

**Model**	**Accuracy**	**Precision**	**F-measure**	**G-means**	**Time(s)**
NM-CNN3	89.6	82.1	88.6	90.3	661
Stacking	86.4	77.3	85.6	87.3	1450
Weighted1	92.1	86.0	91.2	92.7	1721
Voting1	90.4	82.6	89.6	91.2	1723
Weighted2	92.3	86.1	91.5	93.0	7841
Voting2	90.9	83.4	90.1	91.7	7953
Weighted3	**93.2**	**87.4**	**92.4**	**93.8**	8514
Voting3	92.7	86.5	91.9	93.4	8540
Weighted4	93.0	87.0	92.2	93.6	19619
Voting4	91.9	85.0	91.2	92.7	19431

## Discussion

The performance comparative results between the proposed ensemble CNN model and 3 standard classifiers, namely Linear Discriminant Analysis (LDA; Sanguansat et al., 2006), Naive Bayes (NB; Friedman et al., 1997) and Step Discriminant Analysis (SDA), are given in Table 10. The input feature matrix (102^*^10) was first concatenated into a column vector (1020*1). Then the PCA algorithm was used to extract the discriminative features (30^*^1). In LDA, we choose linear distance measurement. In NB, we assume each feature is subject to Gaussian distribution. The default prior probability is the frequency that each class appears. The proposed ensemble model achieved the best classification performance on all the four evaluation indices. For example, it achieved the best classification accuracy of 93.8%, which is 30.7, 34.8, and 9.3% higher than LDA, NB, and SDA classifier, respectively in Case 1. Similarly, it achieved the best classification accuracy of 93.2%, which is 39.3, 40.3, and 18% higher than other three conventional (shallow) classifiers in Case 2. The significant performance enhancement of deep CNN model is due to the fact that its complex structure enables it to extract more effective features.

**Table 10:** Performance comparison of 4 types of classifiers: (a) Case 1; (b) Case 2.

**Method**	**Accuracy**	**Precision**	**F-measure**	**G-mean**
**Case 1**				
Proposed	**93.8**	**89.4**	**92.9**	**94.2**
LDA	63.1	52.7	62.3	64.2
NB	59.0	49.2	59.0	59.5
SDA	84.5	76.6	82.8	85.2
**Case 2**				
Proposed	**93.2**	**87.4**	**92.4**	**93.8**
LDA	53.9	45.2	55.0	54.7
NB	52.8	44.6	53.6	54.2
SDA	75.1	63.7	74.8	76.5

Nevertheless, it should be noted that the proposed method still has the following limitations:

1. A more systematic method or at least guideline for selecting the appropriate parameters (i.e., the number of kernels and the learning rate parameter), other than the heuristic method, should be developed. The weights in the ECNN are determined based on the rankings of CNNs, but the best weighs must be adjusted adaptively according to the ensemble results.
2. The machine learning techniques employed for MWL recognition consist of two stages: feature extraction and classifier design. It is usually a time-consuming (inefficient) trial-and-error procedure to determine the most appropriate feature extraction and classification methods. Furthermore, the machine learning model constructed, e.g., Artificial Neural Network (ANN), is subject-specific. The learned parameters are optimal for one subject, but usually not so for another. This may limit the practicability of the proposed machine learning techniques.
3. The machine learning based classifier is not readily interpretable (or transparent), which would reduce its applicability in applied environments.

## Conclusion and future work

In this paper, we proposed an ECNN framework to solve the MWL classification problem. We examined five CNN models that differ in network depth and convolution kernels. It was found that the deeper CNN model with the small convolutional kernels leads to improved classification performance. Furthermore, we compared four different parameter optimization algorithms for each of the five CNN models. The NM-CNN3 was shown to have the best classification accuracy among the 20 candidate base models. The ECNN model was constructed to further improve classification performance. Extensive comparative results demonstrated that the proposed ECNN framework can effectively bolster the 4- (or 7-) class MWL classification robustness and accuracy.

Along this line of research, future research directions, aimed at overcoming the inherent limitations of the present investigation pointed out in Section Discussion, may include:

1. An adaptive ensemble learning strategy would be proposed and validated.
2. To facilitate the real-world applications, a generic (i.e., subject-independent or cross-subject) machine learning based classifier model needs to be built to accommodate a group of subjects with similar psychophysiological characteristics. This is quite important for online (or real-time) MWL classification.
3. In order to achieve a better balance between the classification accuracy and the interpretability of the classification results, we need to develop fuzzy-rule-based MWL classifier.

## Ethics statement

This study was carried out in accordance with the recommendations of the Guidelines for Research Experiments Involving Human or Animal Participants, Ethics Committee of East China University of Science and Technology, with written informed consent from all subjects. All subjects gave written informed consent in accordance with the Declaration of Helsinki. The protocol was approved by the Ethics Committee of East China University of Science and Technology.

## Author contributions

JZ supervised the whole study including the data analysis methods and procedure and wrote and finalized the submitted manuscript, SL performed data analysis and wrote some of the first draft paper, and RW provided support to the experimental work.

### Conflict of interest statement

The authors declare that the research was conducted in the absence of any commercial or financial relationships that could be construed as a potential conflict of interest.

## References

[B1] Anderson C. W. Devulapalli S. V. Stolz E. A. (1995). Determining mental state from EEG signals using parallel implementations of neural networks. Sci. Program. 4, 171–183. 10.1155/1995/603414

[B2] Bengio Y. Boulanger-Lewandowski N. Pascanu R. (2013). Advances in Optimizing Recurrent Networks, in IEEE International Conference on Acoustics, Speech and Signal Processing (ICASSP) (Vancouver, BC), 8624–8628.

[B3] Bindewald J. M. Miller M. E. Peterson G. L. (2014). A function-to-task process model for adaptive automation system design. Int. J. Hum. Comput. Stud. 72, 822–834. 10.1016/j.ijhcs.2014.07.004

[B4] Burke D. P. Kelly S. P. de Chazal P. Reilly R. B. Finucane C. (2005). A parametric feature extraction and classification strategy for brain-computer interfacing. IEEE Trans. Neural. Syst. Rehabil. Eng. 13, 12–17. 10.1109/TNSRE.2004.841881 15813401

[B5] Kingma D. P. Ba J. (2015). Adam: a method for stochastic optimization, in 3rd International Conference for Learning Representations (San Diego, CA), 1–13.

[B6] Friedman N. Geiger D. Goldszmidt M. (1997). Bayesian network classifiers. Mach. Learn. 29, 131–163. 10.1023/A:1007465528199

[B7] Hollender N. Hofmann C. Deneke M. Schmitz B. (2010). Integrating cognitive load theory and concepts of human–computer interaction. Comput. Hum. Behav. 26, 1278–1288. 10.1016/j.chb.2010.05.031

[B8] Ke Y. Qi H. He F. Liu S. Zhao X. Zhou P.. (2014). An EEG-based mental workload estimator trained on working memory task can work well under simulated multi-attribute task. Front. Hum. Neurosci. 8:703. 10.3389/fnhum.2014.00703 25249967 PMC4157541

[B9] Khan M. E. Babanezhad R. Lin W. Schmidt M. Sugiyama M. (2016). Faster stochastic variational inference using proximal-gradient methods with general divergence functions, in Proceedings of the 32nd Conference on Uncertainty in Artificial Intelligence (Jersey City, NJ), 319–328.

[B10] Kivikangas J. M. Chanel G. Cowley B. Ekman I. Salminen M. Järvelä S. (2011). A review of the use of psychophysiological methods in game research. J. Gaming Virtual Worlds 3, 181–199. 10.1386/jgvw.3.3.181_1

[B11] Krizhevsky A. Sutskever I. Hinton G. E. (2012). Imagenet classification with deep convolutional neural networks. Adv. Neural. Inf. Process. Syst. 25, 1097–1105.

[B12] Lin W. Zhang J. Yin Z. (2013). Instantaneous mental workload level recognition by combining kernel fisher discriminant analysis and Kernel Principal Component Analysis, in 32nd Chinese Control Conference (CCC) (Xi'an), 3607–3612.

[B13] LeCun Y. Huang F. J. Bottou L. (2004). Learning methods for generic object recognition with invariance to pose and lighting, in Proceedings of the 2004 IEEE Computer Society Conference on Computer Vision and Pattern Recognition (Washington, DC), 97–104. 10.1109/CVPR.2004.1315150

[B14] Mahfouf M. Zhang J. Linkens D. A. Nassef A. Nickel P. Hockey G. R. J. (2007). Adaptive fuzzy approaches to modelling operator functional states in a human-machine process control system, in 2007 IEEE International Fuzzy Systems Conference (London), 1–6.

[B15] Mirowski P. Madhavan D. LeCun Y. Kuzniecky R. (2009). Classification of patterns of EEG synchronization for seizure prediction. Clin. Neurophysiol. 120, 1927–1940. 10.1016/j.clinph.2009.09.002 19837629

[B16] Noel J. B. Bauer K. W. Lanning J. W. (2005). Improving pilot mental workload classification through feature exploitation and combination: a feasibility study. Comp. Operations Res. 32, 2713–2730. 10.1016/j.cor.2004.03.022

[B17] Ozcift A. Gulten A. (2011). Classifier ensemble construction with rotation forest to improve medical diagnosis performance of machine learning algorithms. Comput. Methods Programs Biomed. 104, 443–451. 10.1016/j.cmpb.2011.03.018 21531475

[B18] Padilha C. A. D. A. Barone D. A. C. Neto A. D. D. (2016). A multi-level approach using genetic algorithms in an ensemble of least squares support vector machines. Knowl. Based Syst. 106, 85–95. 10.1016/j.knosys.2016.05.033

[B19] Parasuraman R. Sheridan T. B. Wickens C. D. (2000). A model for types and levels of human interaction with automation. IEEE Trans. Syst. Man Cybern. Part A 30, 286–297. 10.1109/3468.844354 11760769

[B20] Parasuraman R. Wilson G. F. (2008). Putting the brain to work: neuroergonomics past, present, and future. Hum. Factors 50, 468–474. 10.1518/001872008X288349 18689055

[B21] Pfurtscheller G. Müller-Putz G. R. Schlögl A. Graimann B. Scherer R. Leeb R.. (2016). 15 years of BCI research at Graz University of Technology: current projects. IEEE Trans. Neural Syst. Rehabil. Eng. 14, 205–210. 10.1109/TNSRE.2006.875528 16792295

[B22] Roth H. Lu L. Liu J. Yao J. Seff A. Cherry K.. (2015). Improving computer-aided detection using convolutional neural networks and random view aggregation. IEEE Trans. Med. Imaging 35, 1170–1181. 10.1109/TMI.2015.2482920 26441412 PMC7340334

[B23] Sanguansat P. Asdornwised W. Jitapunkul S. Marukatat S. (2006). Two-dimensional linear discriminant analysis of principle component vectors for face recognition. IEICE Trans. Inform. Syst. 89, 2164–2170. 10.1093/ietisy/e89-d.7.2164

[B24] Simard P. Y. Steinkraus D. Platt J. C. (2003). Best practices for convolutional neural networks applied to visual document analysis, in 7th International Conference on Document Analysis and Recognition (Edinburgh), 958–963.

[B25] Szegedy C. Liu W. Jia Y. Sermanet P. Reed S. Anguelov D. (2015). Going deeper with convolutions, in IEEE Conference on Computer Vision and Pattern Recognition (CVPR) (Boston, MA), 1–9.

[B26] Tsai A. C. Luh J. J. Lin T. T. (2015). A novel STFT-ranking feature of multi-channel EMG for motion pattern recognition. Expert Syst. Appl. 42, 3327–3341. 10.1016/j.eswa.2014.11.044

[B27] Varsta M. Heikkonen J. Mourino J. (2000). Evaluating the performance of three feature sets for brain-computer interfaces with an early stopping MLP committee, in 15th International Conference on Pattern Recognition (Barcelona), 2907–2910.

[B28] Yildiz A. Akin M. Poyraz M. Kirbas G. (2009). Application of adaptive neuro-fuzzy inference system for vigilance level estimation by using wavelet-entropy feature extraction. Expert Syst. Appl. 36, 7390–7399. 10.1016/j.eswa.2008.09.003

[B29] Yin Z. Zhang J. (2014). Identification of temporal variations in mental workload using locally-linear-embedding-based EEG feature reduction and support-vector-machine-based clustering and classification techniques. Comput. Methods Programs Biomed. 115, 119–134. 10.1016/j.cmpb.2014.04.011 24821400

[B30] Zhang J. W. Zheng C. X. Xie A. (2000). Bispectrum analysis of focal ischemic cerebral EEG signal using third-order recursion method. IEEE Trans. Biomed. Eng. 47, 352–359. 10.1109/10.827296 10743777

[B31] Zhou W. Gotman J. (2009). Automatic removal of eye movement artifacts from the EEG using ICA and the dipole model. Prog. Nat. Sci. 19, 1165–1170. 10.1016/j.pnsc.2008.11.013

